# Functional characterization of thiolase-encoding genes from *Xanthophyllomyces dendrorhous* and their effects on carotenoid synthesis

**DOI:** 10.1186/s12866-016-0893-2

**Published:** 2016-11-21

**Authors:** Nicole Werner, Melissa Gómez, Marcelo Baeza, Víctor Cifuentes, Jennifer Alcaíno

**Affiliations:** Departamento de Ciencias Ecológicas y Centro de Biotecnología, Facultad de Ciencias, Universidad de Chile, Las Palmeras 3425, Casilla 653, Ñuñoa, Santiago, Chile

**Keywords:** Thiolase, Mevalonate, Astaxanthin, Sterols, Carotenoids, Functional complementation

## Abstract

**Background:**

The basidiomycetous yeast *Xanthophyllomyces dendrorhous* has been described as a potential biofactory for terpenoid-derived compounds due to its ability to synthesize astaxanthin. Functional knowledge of the genes involved in terpenoid synthesis would create opportunities to enhance carotenoid production. A thiolase enzyme catalyzes the first step in terpenoid synthesis.

**Results:**

Two potential thiolase-encoding genes were found in the yeast genome; bioinformatically, one was identified as an acetyl-CoA C-acetyltransferase (*ERG10*), and the other was identified as a 3-ketoacyl Co-A thiolase (*POT1*). Heterologous complementation assays in *Saccharomyces cerevisiae* showed that the *ERG10* gene from *X. dendrorhous* could complement the lack of the endogenous *ERG10* gene in *S. cerevisiae*, thereby allowing cellular growth and sterol synthesis. *X. dendrorhous* heterozygous mutants for each gene were created, and a homozygous *POT1* mutant was also obtained. This mutant exhibited changes in pigment composition and higher *ERG10* transcript levels than the wild type strain.

**Conclusions:**

The results support the notion that the *ERG10* gene in *X. dendrorhous* is a functional acetyl-CoA C-acetyltransferase essential for the synthesis of mevalonate in yeast. The *POT1* gene would encode a functional 3-ketoacyl Co-A thiolase that is non-essential for cell growth, but its mutation indirectly affects pigment production.

**Electronic supplementary material:**

The online version of this article (doi:10.1186/s12866-016-0893-2) contains supplementary material, which is available to authorized users.

## Background

Isoprenoids are widely distributed in nature with multiple functions due to their structural diversity and represent an important resource for the biotechnology industry with uses ranging from aroma and flavor enhancers (terpenes) to anticarcinogenic molecules (taxol) [1]. All isoprenoids originate from isopentenyl pyrophosphate (IPP), containing five carbon units, which in most organisms is obtained through the mevalonate (MVA) pathway or the methyl-D-erythritol-4-phosphate (MEP) pathway, the latter of which occurs in plant plastids, algae and some bacteria [2].

Condensation between two acetyl-CoA molecules to form acetoacetyl-CoA has been recognized as the first step in the mevalonate pathway in eukaryotes [3]. This reaction is catalyzed through a non-decarboxylative Claisen-type condensation by an enzyme that belongs to the thiolase protein family known as acetyl-CoA C-acetyltransferase (ACAT) [4, 5]. The thiolase protein family comprises enzymes that have different subcellular localizations and expression patterns, depending on the pathway with which they are associated; in yeast, enzymes with cytoplasmic locations are mevalonate pathway related enzymes (ACAT), and 3-ketoacyl-CoA thiolase (ACAA), which is involved in the β-oxidation of fatty acids, is found in peroxisomal locations [6, 7].

The second step in mevalonate biosynthesis involves the addition of a third acetyl-CoA molecule to form 3-hydroxy-3-methylglutaryl-CoA (HMG-CoA) by the HMG-CoA synthase (HMGS) enzyme, followed by the conversion of HMG-CoA to mevalonic acid via HMG-CoA reductase (HMGR), which is the most studied step of the mevalonate pathway and has been defined as the rate-limiting step in sterol biosynthesis in eukaryotes [8]. In *Saccharomyces cerevisiae,* two HMGR encoding genes have been identified, *HMG1* and *HMG2,* with *HMG1* contributing the majority of the enzymatic activity in the pathway [9]; however, only one ACAT encoding gene (*ERG10*) has been reported in this yeast [10]. The concluding steps in the mevalonate pathway include two sequential phosphorylation reactions performed by the enzymes mevalonate kinase and phosphomevalonate kinase, respectively, and a final decarboxylation step catalyzed by phosphomevalonate decarboxylase to produce IPP [11], which is the precursor of the wide variety of isoprenoid compounds and derivatives, including secondary metabolites of commercial value.

The basidiomycetous yeast *Xanthophyllomyces dendrorhous* has been extensively studied for its ability to produce large amounts of isoprenoids, primarily astaxanthin (3,3′-dihydroxy-b, b-carotene-4,4′-dione), a red carotenoid used as a feeding additive in aquaculture, making it appealing to the biotechnological industry. Most genetic studies with this yeast have focused on the characterization of genes involved in carotenogenesis [12, 13] and genes involved in the biosynthesis of carotenoids precursors [14]. It has been shown that astaxanthin production in *X. dendrorhous* is favored when cultures are supplemented with mevalonate [15] or in strains that have higher *HMGR* transcript levels [16]. These results reflect the critical role of the MVA pathway in carotenogenesis in *X. dendrorhous*, highlighting the importance of studying the MVA pathway and identifying the genes that are directly involved in the synthesis of IPP.

Regarding the *ERG10* gene of *X. dendrorhous,* a possible ACAT encoding gene was recently identified in a genomic study of this yeast [17], and its overexpression in *X. dendrorhous* led to an increase in total carotenoid production [18]. In the present study, another possible *X. dendrorhous* thiolase-encoding gene is identified, and additional functional evidence for the previously reported ACAT encoding gene and for the newly characterized ACAA encoding gene in this yeast is provided*.*

## Results and discussion

### Bioinformatic characterization and expression analysis

Gene identification was accomplished by running a local BLASTp search in the program CLC Genomics Workbench through the *X. dendrorhous* genomic and transcriptomic databases [19], using related *ERG10* sequences obtained from the GenBank database as queries. Two potential *ERG10* sequences were obtained and designated *ERG10A* and *ERG10B*. The *ERG10A* gene is composed of 7 introns and 8 exons, and its cDNA has a length of 1,296 bp encoding a deduced protein of 431 amino acids with a total size of 44.38 kDa, while the *ERG10B* gene contains 5 introns and 6 exons, and its cDNA has a length of 1,212 bp encoding a 42.01-kDa deduced protein of 403 amino acids.

Within the thiolase family, degradative and biosynthetic enzymes share the same catalytic mechanism in which the catalytic site is conformed by four loops, each carrying conserved residues at the active site [4]. These residues correspond to Cys-125, Asn-357, His-389 and Cys-417 in the deduced thiolase encoded by the *ERG10A* gene and Cys-101, Asn-326, His-358 and Cys-388 in the thiolase deduced from *ERG10B.* Two reactions occur in the catalytic site of this enzyme family; first a conserved highly reactive Cys residue (Cys-89 in the *Zoogloea ramigera* biosynthetic thiolase [PDB: 1 dm3]) is acetylated, while a second Cys (Cys-378 in the *Z. ramigera* biosynthetic thiolase) protonates the CoA leaving group. The second reaction involves condensation, in which the negatively charged Cys-378 deprotonates a second acetyl-CoA, to which the acetyl moiety of Cys-89 is transferred [4, 5]. According to bioinformatic analyses, both putative *X. dendrorhous* thiolases contain all of the catalytic site amino acids, but no further information regarding their catalytic activity could be obtained.

**Fig. 1:**
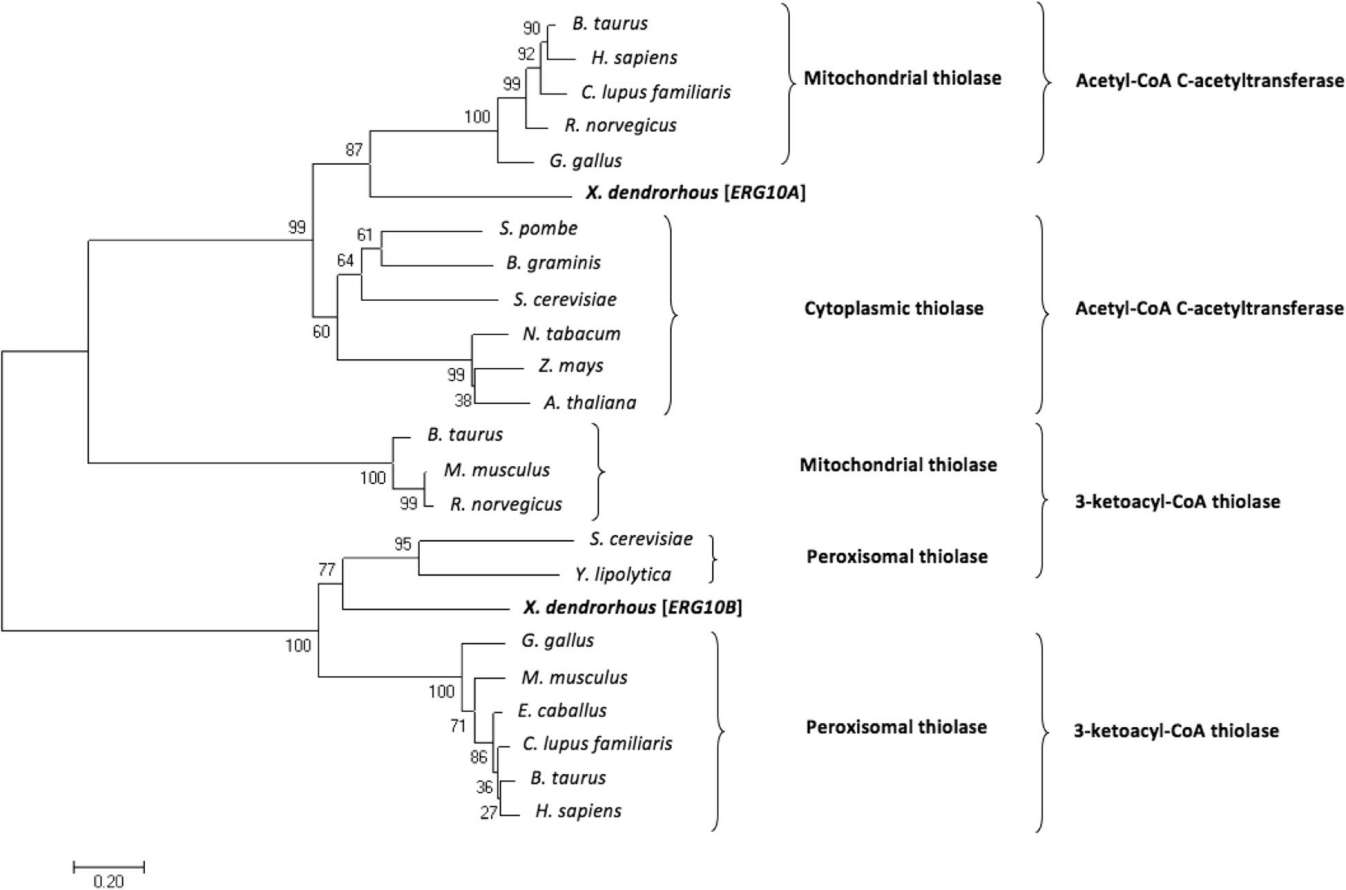
Phylogenetic tree of *ERG10A* and *ERG10B* thiolases from *X. dendrorhous* compared to other organisms. The unrooted tree was created in MEGA 6.0 using the neighbor-joining method [20] with the following amino acid sequences. Mitochondrial thiolase/acetyl-CoA C-acetyltransferase: *B. taurus* [NP_001039540.1], *H. sapiens* [BAA01387.1], *C. lupus familiaris* [XP_546539.2], *R. norvegicus* [NP_058771.2], *G. gallus* [NP_001264708.1]. Cytoplasmic thiolase/acetyl-CoA C-acetyltransferase: *S. pombe* [Q9UQW6.1], *B. graminis f. sp. tritici 96224* [EPQ61678.1], *S. cerevisiae* [P41338.3], *N. tabacum* [AAU95618.1], *Z. mays* [NP_001266315.1], *A. thaliana* [Q9FIK7.1]. Mitochondrial thiolase/3-ketoacyl-CoA thiolase: *B. taurus* [NP_001030419.1], *M. musculus* [NP_803421.1], *R. norvegicus* [NP_569117.1]. Peroxisomal thiolase/3-ketoacyl-CoA thiolase: *S. cerevisiae* [CAA37472.1], *Y. lipolytica* [Q05493.1], *G. gallus* [NP_001184217.1], *M. musculus* [NP_570934.1], *E. caballus* [XP_001488609.1], *C. lupus familiaris* [XP_534222.2]. *B. taurus* [NP_001029491.1], *H. sapiens* [NP_001598.1]. *X. dendrorhous* [*ERG10A*]: thiolase encoded by the *ERG10A* gene. *X. dendrorhous* [*ERG10B*]: thiolase encoded by the *ERG10B* gene. Accession numbers are given in parentheses. Numbers at each node indicate the percentage support for a specific node after 1,000-replica bootstrap analysis

To determine the possible function of the thiolase encoded by each gene, a phylogenetic tree was created using thiolase amino acid sequences described as having different functions: acetyl-CoA C-acetyltransferase or 3-ketoacyl-CoA thiolase. For multiple alignments, the program ClustalW 2.1 was used and a phylogenetic tree was built using the Neighbor Joining method with a 1,000-replica bootstrap with MEGA6 software [20]. As shown in Fig. 1, the thiolase encoded by the *ERG10A* gene groups with acetyl-CoA C-acetyltransferases, and the thiolase encoded by *ERG10B* groups with 3-ketoacyl-CoA thiolases. The multiple alignment used to generate this tree is shown in Additional file 1: Figure S1, where Cys-141, Asn-384, His-416 and Cys-447 correspond to the conserved active site residues. This result suggests that *ERG10A* most likely encodes the thiolase involved in the mevalonate pathway and that *ERG10B* is the thiolase involved in the β-oxidation of fatty acids in *X. dendrorhous*. Moreover, the *ERG10A* nucleotide sequence has a 98% identity with 91% coverage compared to the *X. dendrorhous acaT* reported nucleotide sequence available at GenBank [AB919149] [18].

**Fig. 2:**
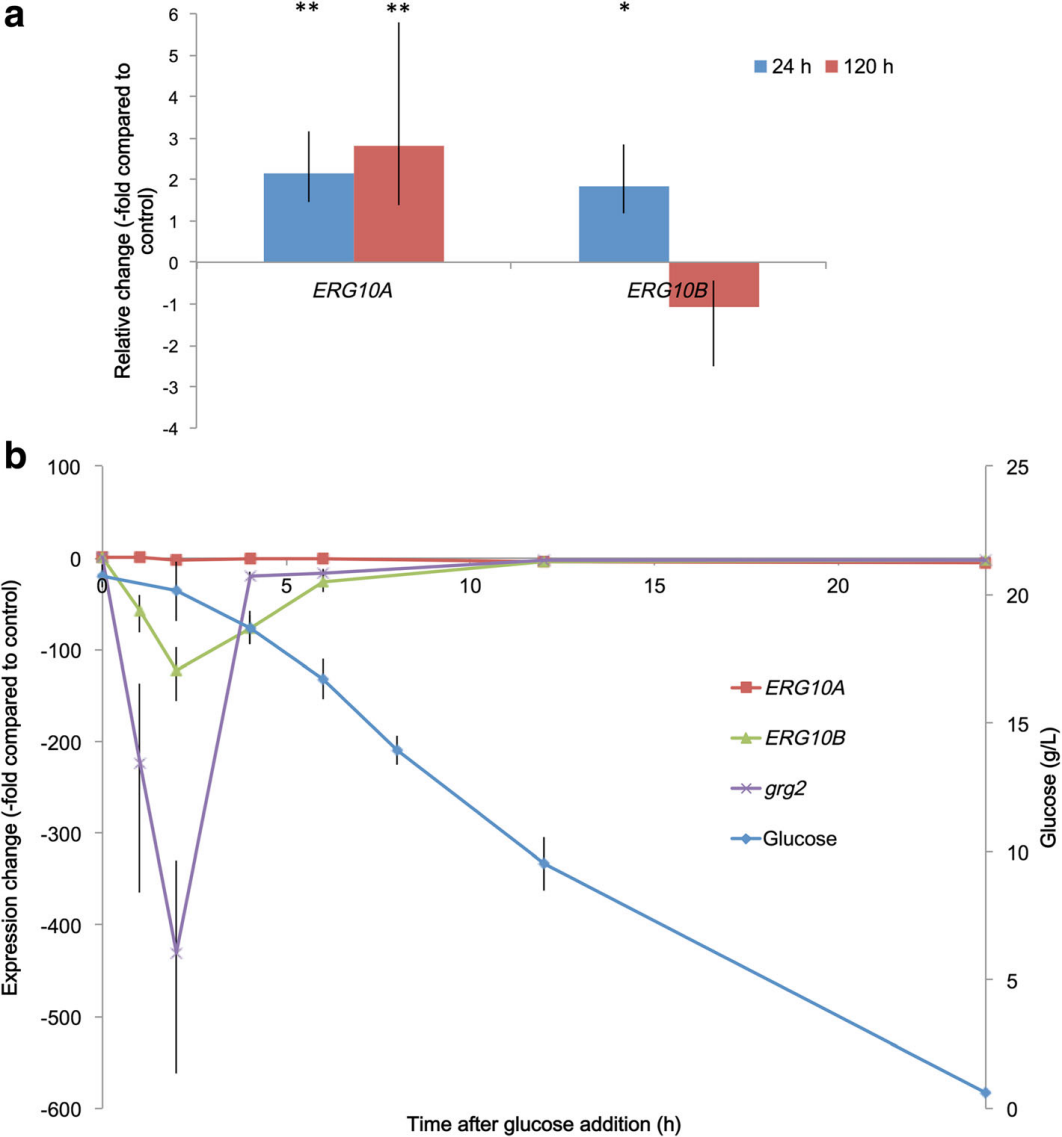
*ERG10A* and *ERG10B* relative transcript levels. **a** In strain 385-*cyp61* ^(−/−)^, which does not produce ergosterol. Level of change was determined by comparison to wild type UCD 67–385 (control)*.* Cultures were grown in YM liquid media for 24 h (blue) or 120 h (red). Each bar represents an average of three independent cultures. Black lines indicate standard deviation. *p < 0.05, **p < 0.01. **b** Effect of glucose addition. Gene expression kinetics and glucose concentration were quantified in strain UCD 67–385 after adding glucose (20 g/l final concentration). Error bars correspond to the standard deviation (*n* = 3). The negative values on the y-axis denote decrease relative to control

To identify possible binding sites for transcriptional regulators, the promoter regions, which were considered to comprise 1,000 bp upstream of the first ATG codon of each gene, were analyzed using JASPAR (http://jaspar.genereg.net/) and TBfind (http://tfbind.hgc.jp/). Several genes involved in the MVA pathway of *Schizosaccharomyces pombe* have been shown to be regulated by ergosterol levels through the Sterol Regulatory Element Binding Protein (SREBP), which has been described as a major regulator of genes related to the synthesis of cellular sterols [21]. In the promoter analysis, one possible SRE element was identified upstream of each *ERG10* gene-encoding region, suggesting possible regulation through SREBP. To gain further insight, the relative transcript levels of both genes were analyzed in a *X. dendrorhous* mutant strain (385-*cyp61* ^(−/−)^) that does not produce ergosterol but accumulates other sterols and demonstrates up-regulation of the *HMGR* gene involved in the MVA pathway in relation to the wild type strain UCD 67–385 [16]. Transcript levels for the *ERG10A* and *ERG10B* genes were evaluated in both strains after 24 h (exponential stage) and 120 h (stationary stage) of culture (Fig. 2a). At both time points, the transcript levels of *ERG10A* were higher in the mutant strain in relation to the wild type strain. Maximum differences were observed for the *ERG10A* transcript levels after 120 h of culture (approximately 3-fold higher in the mutant strain in relation to wild type). For *ERG10B,* a significant change in transcript levels was observed only after 24 h. In the study made by Loto et al. using the same strain and growth conditions [16], transcripts for the *HMGR* gene were measured. The change in the expression of this gene was 23 times higher in the strain that doesn’t produce ergosterol compared to wild type strain. As the SREBP pathway has been described as a regulatory mechanism that acts upon genes related to sterol biosynthesis [21], the change could be attributed to a SREBP-like mechanism. Compared to these results, the change in expression of *ERG10A* is minor and cannot be attributed to SREBP *a priori*, although the gene contains potential SRE sites in its promoter region. Further experiments should be performed in the future to identify if there is a functional SREBP-like mechanism in *X. dendrorhous* that may be regulating the aforementioned genes.

According to Marcoleta et al. [22], carotenogenesis in *X. dendrorhous* is repressed by glucose. Moreover, the *MIG1* gene encoding the catabolic repressor Mig1, which mediates transcriptional glucose-dependent repression in other yeasts, was recently described and its mutation alleviated the glucose-mediated repression of carotenogenesis in *X. dendrorhous* [23]. Considering these findings, the possible glucose-dependent regulation of *ERG10A* and *ERG10B* gene expression was studied to evaluate whether catabolite repression could act over pathways upstream of carotenogenesis. For this, the wild type *X. dendrorhous* strain was cultured in YM media without glucose supplementation until the stationary phase of growth was reached; then, the culture was divided into two flasks: one was supplemented with glucose to a final concentration of 20 mg/ml and the other was left as a control without glucose supplementation. Cultures were incubated at 22 °C with constant agitation, and samples were taken after 1, 2, 4, 6, 12 and 24 h of treatment to extract total RNA and evaluate *ERG10A* and *ERG10B* transcript levels by RT-qPCR (Fig. 2b). The *ERG10A* gene transcript levels did not show any significant changes after the addition of glucose, but they were reduced approximately 5-fold compared to the control when glucose consumption began, which could be attributable to the by-products of glucose metabolism affecting *ERG10A* expression. In contrast, *ERG10B* transcript levels decreased approximately 120-fold when glucose was added to the culture compared to the untreated control, in a similar manner to the glucose repressed gene *grg2* [GenBank: JN043364], used as a glucose repression control (Fig. 2b) [22]. However, this decrease was only temporary as the transcript levels normalized to control levels when the glucose in the media was consumed. Considering these results, only *ERG10B* is repressed by glucose in a manner similar to catabolite repression. Glucose repression, mediated by the transcription factors UME6, ABF1 and RP-A, has been reported previously for the *S. cerevisiae* gene *POT1/FOX3*, which encodes the yeast ACAA thiolase [24]. This evidence also supports the notion that *ERG10B* could indeed encode the ACAA thiolase in *X. dendrorhous*.

The results presented above support the idea that *ERG10A* encodes an acetyl-CoA C-acetyltransferase and *ERG10B* encodes a 3-ketoacyl-CoA thiolase, each participating in a different metabolic pathway (Additional file 2: Figure S2). Thus, hereafter each gene denomination was assigned according to the gene name given in *S. cerevisiae*, *ERG10* to *ERG10A* and *POT1* to *ERG10B*, and the sequences were uploaded to the GenBank database [KX267759 and KX26758, respectively].

### Functional complementation in *S. cerevisiae*

To functionally assess the bioinformatic results, we performed heterologous expression of each gene cDNA in *S. cerevisiae* for complementation assays. For this analysis the *S. cerevisiae* diploid strain Meyen ex E.C Hansen YPL028W BY4743 (*Sc-ERG10/erg10*), a heterozygous mutant for the *ERG10* gene, was acquired from the ATCC collection.

The *S. cerevisiae ERG10* gene and the cDNAs corresponding to the two potential *X. dendrorhous* thiolase-encoding genes (*ERG10* and *POT1)* were inserted into the *S. cerevisiae* expression vector YEpNP (Table 1) and used to independently transform *Sc-ERG10/erg10.* From each transformation, two random colonies were selected and analyzed by PCR to confirm the presence of the tested gene in the plasmid.

**Table 1:** Strains and plasmids used in this work

Strain/Plasmid	Genotype or relevant features	Reference
Strain:		
*X. dendrorhous*		
UCD 67–385	Wild type, diploid strain.	ATCC 24230
385-*cyp61*(−/−)	Homozygote transformant derived from UCD 67–385 with both *CYP61* alleles interrupted, one with a hygromycin B resistance cassette and the other with a zeocin resistance cassette.	[16]
385-*erg10*(+/−)	Heterozygous mutant of UCD 67–385 with one allele of *ERG10* replaced by a hygromycin B resistance cassette.	This work
385-*pot1*(+/−)	Heterozygous mutant of UCD 67–385 with one allele of *POT1* replaced by a hygromycin B resistance cassette.	This work
385-*pot1*(−/−)	Homozygous mutant of UCD 67–385 with both *POT1* alleles replaced by a hygromycin B resistance cassette.	This work
385-*ERG10*	Heterozygote transformant derived from UCD 67–385 containing an additional *ERG10* allele and a hygromycin B resistance cassette integrated at *locus int*.	This work
385-*POT1*	Heterozygote transformant derived from UCD 67–385 containing an additional *POT1* allele and a hygromycin B resistance cassette integrated at *locus int*.	This work
385-Vexp2	Heterozygote transformant derived from UCD 67–385 containing an empty over-expressing cassette (without an inserted ORF) and a hygromycin B resistance cassette integrated at *locus int*.	This work
*S. cerevisiae*		
s288C	*MATα, SUC2, gal2, mal, mel, flo1, flo8-1, hap1, ho, bio1, bio6*	[38]
ATCC 4022800 (Sc-ERG10/erg10)	*MATa/MATalpha his3delta1/his3delta1 leu2delta0/leu2delta0 lys2delta0/+ met15delta0/+ ura3delta0/ura3delta0 deltaERG10*	[39]
Sc-ERG10sc	Strain Sc-ERG10/erg10 carrying plasmid YEpNP-10sc.	This work
Sc-cERG10xd	Strain Sc-ERG10/erg10 carrying plasmid YEpNP-c10Xd.	This work
Sc-cPOT1xd	Strain Sc-ERG10/erg10 carrying plasmid YEpNP-cPOT1Xd.	This work
Plasmid:		
pBluescript SK- (pBS)	ColE1 replication origin, Amp^R^, LacZ for blue-white colony screening.	Stratagene
pBS-PT-ERG10xd	pBS with 620 bp of DNA upstream of *ERG10* gene and 412 bp downstream with a *Sma*I site between them.	This work
pBS-PT-POT1xd	pBS with 559 bp of DNA upstream of *POT1* gene and 560 bp downstream with a *Sma*I site between them.	This work
pMN-*hph*	Plasmid containing an hygromycin B resistance cassette for *X. dendrorhous.*	[12]
pPHT-Erg10xd	pBS -PT-ERG10Xd with a hygromycin B resistance cassette in the *Sma*I site.	This work
pPHT-POT1xd	pBS-PT-POT1Xd with a hygromycin B resistance cassette in the *Sma*I site.	This work
pPZT-Erg10xd	pBS-PT-ERG10Xd with a zeocin resistance cassette in the *Sma*I site.	This work
YEp-ACT4	pBR322 and 2 micron replication origins, Amp^R^, *LEU2*, promoter *ACT4.*	[32]
YEp-NP	YEpACT4 with a *TDH3* terminator next to pACT4.	[28]
YEpNP-10sc	YEp-NP with *ERG10* DNA from *S. cerevisiae* between p*ACT4* and tTDH3.	This work
YEpNP-c10xd	YEp-NP with *ERG10* from *X. dendrorhous* cDNA between pACT4 and tTDH3.	This work
YEpNP-cPOT1xd	YEp-NP with *POT1 from X. dendrorhous* cDNA between pACT4 and tTDH3	This work
pXdVexp2	*X. dendrorhous* expression vector: pBS bearing the *X. dendrorhous* ubiquitin promoter [GenBank: KJ140285] and GPD terminator [Genbank:Y08366] with a *Bam*HI site between them to insert the gene to express and the hygromycin B cassette for selection, flanked by non-encoding genomic [GenBank: KJ140286] regions to target the construction integration in the genome.	[14]
pXdVexp2-c*ERG10*xd	pXdVexp2 containing the cDNA version of the *ERG10* gene from *X. dendrorhous*	This work
pXdVexp2-c*POT1*xd	pXdVexp2 containing the cDNA version of the *POT1* gene from *X. dendrorhous*	This work

**Fig. 3:**
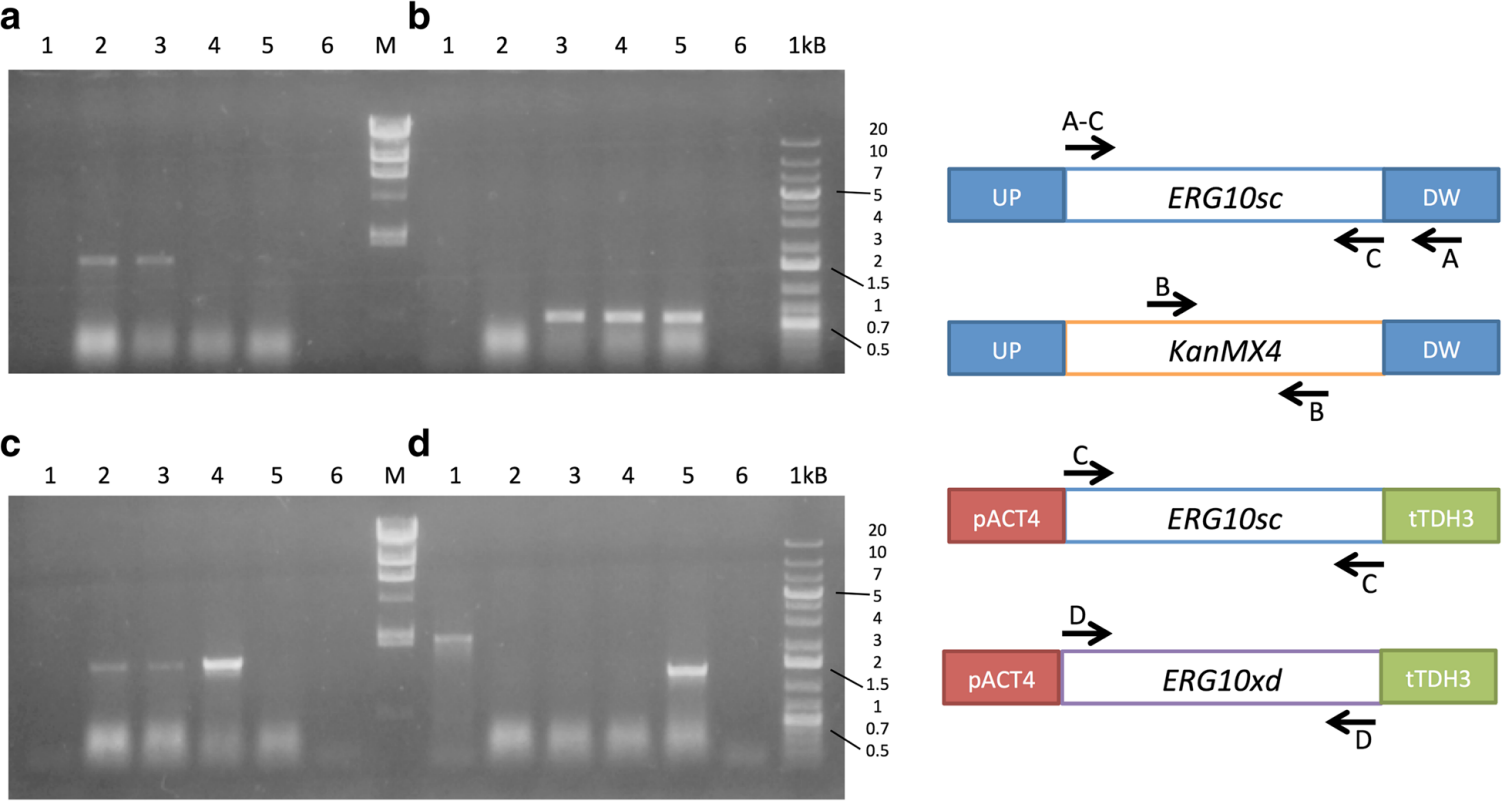
PCR analyses of *S. cerevisiae* haploid strains. *S. cerevisiae* strains Sc-ERG10sc (carrying plasmid YEpNP-10sc) and Sc-cERG10xd (carrying plasmid YEpNP-c10xd) were analyzed by PCR to confirm the expected genotype. As controls, *X. dendrorhous* UCD 67–385 strain (Lane 1), *S. cerevisiae* strain S288c (Lane 2), *S. cerevisiae* diploid strain *Sc-ERG10/erg10* (Lane 3) and a no-template control (Lane 6), were included. *S. cerevisiae* Sc-ERG10sc haploid strain (Lane 4) and *S. cerevisiae* Sc-cERG10xd haploid strain (Lane 5) were analyzed to assess: **a** absence of chromosomal *ERG10* from *S. cerevisiae* (primers erg10scF and erg10scDWR); **b** presence of geneticin resistance cassette in *S. cerevisiae* (primers KanMXF2 and KanMXR2); **c** presence of *ERG10* from *S. cerevisiae* (primers erg10scF and erg10scR); and **d** presence of *ERG10* from *X. dendrorhous* (primers Thio2Fw and Thio2Rv). The molecular size markers Lambda DNA/*Hind*III (Lane M; 23.1, 9.4, 6.6, 4.4, 2.3, 2 and 0.6 kb) and GeneRuler 1 kb Plus (Lane 1kB, band size in kb is indicated) were used. On the right side of the picture, a schematic diagram of the amplification products is included; arrows represent primer sets with a letter indicating in which panel they were used. UP and DOWN (in blue) correspond to chromosomal regions located approximately 300 bp upstream and downstream of the *S. cerevisiae ERG10* gene, respectively, KanMX4 corresponds to the geneticin (G418) resistance module, and pACT4 (in red) and tTDH3 (in green) correspond to promoter and terminator regions in the vector YEpNP, respectively

Acetyl-CoA C-acetyltransferase activity is essential for cell viability [10]. In *S. cerevisiae*, this activity is only performed by the enzyme encoded by *ERG10*, so loss of this gene leads to unviable mutants. To assess if the gene expressed by the plasmid has the specified function, Random Spore Analysis was performed. After sporulation of the transformant strains and asci breaking followed by haploid selection, total DNA was extracted to confirm the lack of the endogenous *ERG10* gene and presence of the complementing gene in the plasmid by PCR analyses (Fig. 3). These could only be confirmed in strains carrying the YEpNP-10sc and YEpNP-c10xd plasmids (corresponding to strains Sc-ERG10sc and Sc-cERG10xd) that contain the *ERG10* genes from *S. cerevisiae* and *X. dendrorhous*, respectively. As shown in Fig. 3a, the chromosomal *ERG10* gene from *S. cerevisiae* was amplified only from strains S288c and Sc-dERG10, as haploid strains do not have this gene; instead, the band corresponding to the KanMX resistance gene was amplified from that locus (Fig. 3b). The *S. cerevisiae ERG10* gene could be fully detected in the control strain S288c, the diploid mutant strain Sc-dERG10 and strain Sc-ERG10Sc, which carry the plasmid harboring this gene (Fig. 3c). A fragment of approximately 1,250 bp was amplified from strain Sc-cERG10xd, which corresponds to the cDNA of gene *ERG10* from *X. dendrorhous (*Fig. 3d); the same primer pair amplified a fragment of approximately 2,200 bp when genomic DNA from strain UCD 67–385 of *X. dendrorhous* was used as template, corresponding to the genomic version of *ERG10*. In the complementation assays with the *X. dendrorhous POT1* gene, no haploid colonies lacking the endogenous *ERG10* gene could be identified, even though more than 500 colonies were analyzed by replica plating and 30 potential candidates were analyzed by PCR.

Heterologous complementation in haploid strains was further analyzed by constructing growth curves (Additional file 3: Figure S3) and performing total sterol quantification/composition analyses. Samples for sterol extraction were recovered after 48 h of growth. The total amounts of sterols recovered from 10-ml samples were 3.3 ± 0.2 (mg/g dry yeast) for strain Sc-*ERG10/erg10*, 4.67 ± 0.01 (mg/g dry yeast) for strain Sc-ERG10sc and 6.4 ± 0.7 (mg/g dry yeast) for strain Sc-cERG10xd. Differences in the amounts of sterols produced between the parental diploid strain and the complemented strains may be attributable to the fact that in the latter, the gene is carried in an expression plasmid from which transcription is not regulated, leading to higher transcription rates.

These results support the notion that *ERG10* indeed encodes the *X. dendrorhous* acetyl-CoA C-acetyltransferase as it complements the *erg10*- mutation in *S. cerevisiae*.

### *ERG10* and *POT1* gene mutations in *X. dendrorhous*

To gain further knowledge regarding the functions and effects on carotenogenesis of the potential thiolase-encoding genes identified in *X. dendrorhous*, deletion mutants were generated. Plasmids pPHT-ERG10xd and pPHT-POT1xd were constructed and linearized to independently transform the diploid wild type strain UCD 67–385 to replace the corresponding genes with a hygromycin B resistance cassette by homologous recombination. PCR analyses were performed to confirm the gene replacement events on the heterozygous mutant strains obtained for genes *ERG10* (385-*erg10* ^(+/−)^) and *POT1* (385-*pot1* ^(+/−)^). To the naked eye, no pigmentation differences between both heterozygous mutant strains and the wild type could be appreciated. This observation was confirmed by pigment extraction and quantification from triplicate samples grown in liquid YM media at 22 °C for three days. The total amount of carotenoids extracted was 191 ± 5 μg/g dry weight for 385-*erg10* ^(+/−)^ and 175 ± 8 μg/g dry weight for 385-*pot1* ^(+/−)^ compared to 177 ± 11 μg/g dry weight from wild-type strain.

Among the properties that differentiate ACAT from ACAA encoding genes is the fact that ACAA null mutants can be obtained [25], whereas ACAT null mutants cannot unless there is more than one gene encoding enzymes with the same activity. We attempted to obtain *X. dendrorhous* double mutants for both strains using the double recombinant method (DRM, [26]). Briefly, the method consists of growing a heterozygous mutant in liquid media with increasing concentrations of the antibiotic corresponding to the selection marker considering that strains that become homozygous (by mitotic recombination) for the antibiotic marker would be able to grow at a higher concentration of the antibiotic than heterozygous strains. Antibiotic concentration was increased until a phenotypic color difference in colonies grown in plates was observed. For 385-*erg10* ^(+/−)^, antibiotic concentration could only be augmented from 15 μg/ml up to 100 μg/ml as higher concentrations inhibited cell growth; however, no differences in color phenotype were observed in the seeded colonies. Growth of strain 385-*pot1* ^(+/−)^ could still be accomplished at the maximum concentration of antibiotic used (400 μg/ml), and seeded colonies began to exhibit a paler phenotype when the antibiotic concentration in liquid media reached 200 μg/ml.

For both heterozygous mutant strain DRM assays, a few colonies selected at the maximum possible antibiotic concentration were randomly chosen and analyzed by PCR to determine if both alleles of the studied genes were lost after the treatment. No homozygous mutants deriving from strain 385-*erg10* ^(+/−)^ were found. Although this is an expected result if *ERG10* encodes the thiolase involved in the MVA pathway, a second approach was attempted to try to obtain a homozygous mutant. A second plasmid for transformation, pPZT-ERG10xd, was constructed and used to transform 385-*erg10* ^(+/−)^; transformant selection was performed using plates supplemented with zeocin and hygromycin B. No colonies resistant to both antibiotics were obtained after several attempts of transformation, suggesting that a homozygous mutant for gene *ERG10* may not be viable as expected considering that *ERG10* is an essential gene.

On the other hand, it was confirmed by PCR analyses that paler colonies deriving from strain 385-*pot1* ^(+/−)^ after applying the DRM had lost both *POT1* alleles (homozygous mutants). For further analyses, only one of the analyzed colonies was used and designated 385-*pot1* ^(−/−)^.

**Fig. 4:**
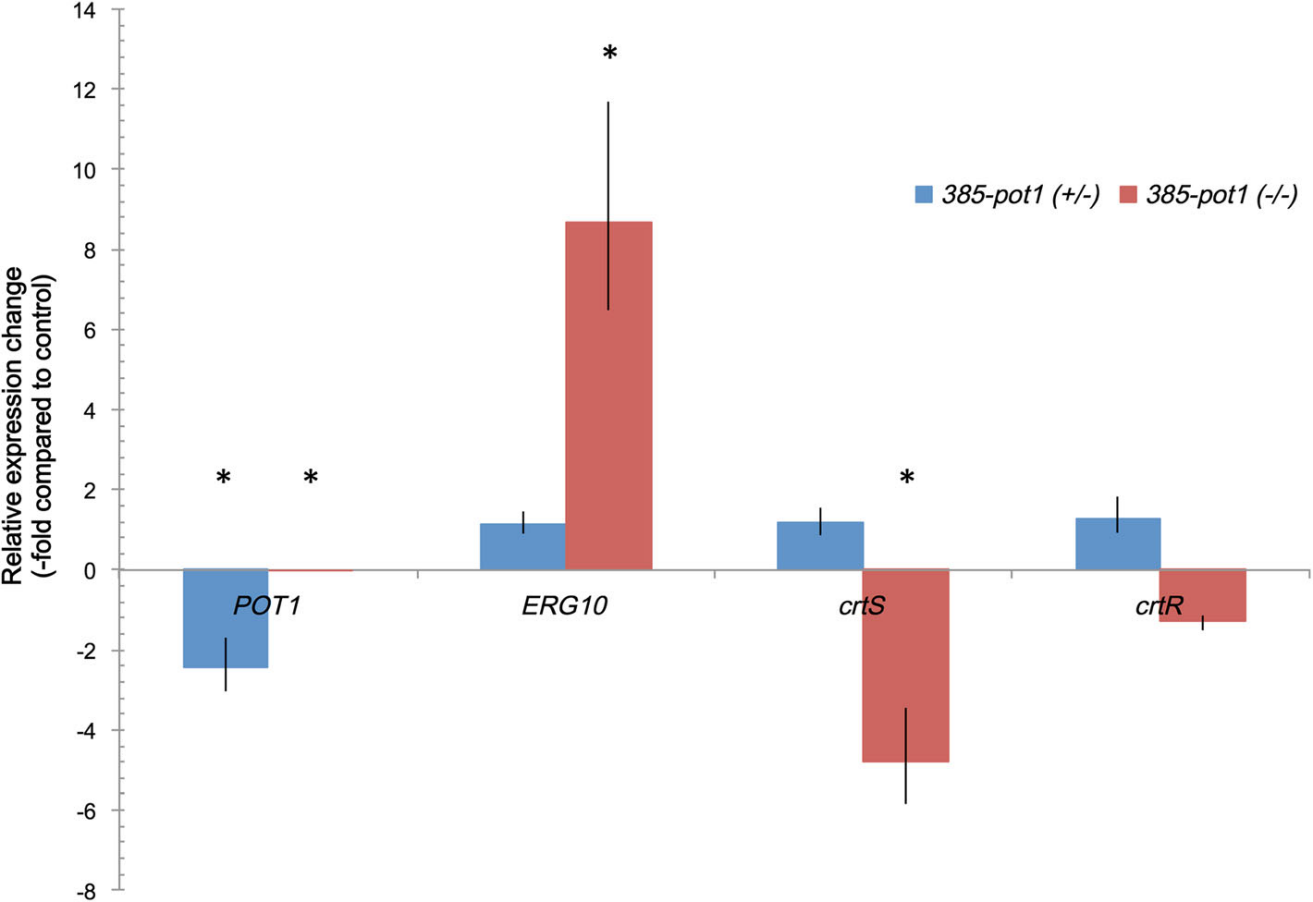
Changes in transcript levels for *POT1* mutant strains. Relative transcript levels of genes *POT1*, *ERG10*, *crtS* and *crtR* of *X. dendrorhous* were analyzed in samples obtained after 96 h of growth in liquid YM media for strains UCD 67–385, 385-*pot1* ^(+/−)^ and 385-*pot1* ^(−/−)^. The results were analyzed using the 2^-ΔΔCt^ method using actin as the normalizer gene and UCD 67–385 as the control strain compared to 385-*pot1* ^(+/−)^ (blue) and 385-*pot1* ^(−/−)^ (red). For *POT1* transcript analysis in strain 385-*pot1* ^(−/−)^, no transcript was detected. Each bar represents an average of three independent samples. Black lines indicate standard deviation. *p < 0.01

To compare phenotypic changes between 385-*pot1* ^(+/−)^, 385-*pot1* ^(−/−)^ mutants and the wild type strain, each was grown in YM media at 22 °C with constant agitation for four days in triplicate. Samples were taken after 96 h of culture (stationary phase of growth) to analyze carotenoid and sterol content and composition. Within the growth curve, no significant differences were observed, either for total sterol or in carotenoid quantification. Carotenoid samples were analyzed by RP-HPLC and, as expected from visual inspection, strain 385-*pot1* ^(−/−)^ showed major differences regarding carotenoid composition in relation to the parental and heterozygous mutant strains, with a reduced proportion of astaxanthin and an increase in carotenogenesis intermediaries (Table 2). These results are similar to what has been previously observed in strains in which genes leading to sterol synthesis and carotenogenic pathways precursors were deleted to obtain heterozygous mutants [14]. In those mutants, decreases in the transcript levels of the genes *crtS* and *crtR*, which control the synthesis of astaxanthin from beta-carotene, were reduced, which could partially explain their differential carotenoid compositions compared to the wild type strain. Considering this background, total RNA was extracted from each sample, and RT-qPCR analyses were performed to compare the relative amounts of transcripts of the genes *ERG10*, *POT1*, *crtS* and *crtR* (Fig. 4). In the 385-*pot1* ^(+/−)^ strain, only the *POT1* transcript showed significant changes, demonstrating half the amount of transcript when compared to the parental strain UCD 67–385. In the null 385-*pot1* ^(−/−)^ mutant, *POT1* transcripts were not detected, confirming that the strain does not have the functional gene. In this strain, the *ERG10* transcript level was increased 8-fold compared to the wild type, suggesting that some compensation for *POT1* gene loss might be occurring. Although ACAA enzymes (encoded by *POT1*) are not directly related to the carotenogenic pathway, they might influence substrate availability as they catalyze the final steps in fatty acid β-oxidation, which could alter the acetyl-CoA pool in the peroxisome that can be transported to the mitochondria for energy production through the TCA cycle [27]. Then, if no acetyl-CoA is obtained from peroxisomal fatty acid β-oxidation, more cytoplasmic acetyl-CoA could be transported to the mitochondria; thus, a higher *ERG10* transcript level could help to ensure the cytoplasmic acetyl-CoA flux towards the MVA pathway to maintain the sterol production necessary for cell replication. Although no significant changes were found in the *crtR* transcript level in the mutant 385-*pot1* ^(−/−)^, the *crtS* transcript level was reduced 5-fold, which could explain, at least in part, the differences observed in carotenoid composition. The *crtS* gene encodes a cytochrome P450 enzyme, and it has been shown that in *X. dendrorhous,* the *crtR* gene that encodes the cytochrome P450 reductase is essential for the synthesis of astaxanthin in this yeast [13]. Then, considering that two cytochrome P450 enzymes are involved in ergosterol biosynthesis [16, 28], the reduced *crtS* transcript levels could be related to reduced carotenogenic activity to maintain the flux towards ergosterol biosynthesis.

**Table 2:** Total sterol and carotenoid content in *X. dendrorhous POT1* mutant strains

Strain	UCD 67–385	385-*pot1*(+/−)	385-*pot1*(−/−)
Total Sterols (mg/g dry weight)	5.7 ± 0.3	4.6 ± 0.2	5.6 ± 0.6
Total Carotenoids (μg/g dry weight)	207.8 ± 21.5	201.5 ± 3.8	174 ± 19.5
% Astaxanthin	82.0 ± 3.4	78.6 ± 0.7	49.4* ± 2.4
% Beta-carotene	2.3 ± 0.7	2.8 ± 0.1	10.0* ± 0.8
% Phoenicoxanthin	8.0 ± 1.3	9.9 ± 0.4	16.5* ± 0.6
% Other carotenoids	7.7 ± 1.7	8.6 ± 0.3	23.0* ± 2.0

Each value corresponds to the average of three independent samples ± standard deviation. Other carotenoids include hydroxy-keto-γ-carotene, hydroxy-equinenone, hydroxy-keto-torulene and canthaxanthin. *p < 0.01, Student’s *T*-test compared to wild type

### *ERG10* and *POT1* gene overexpression in *X. dendrorhous*

To evaluate if overexpression of the studied genes affects the biosynthesis of secondary metabolites, strains that carry an extra copy of each gene were constructed. cDNAs of genes *ERG10* and *POT1* were inserted into the plasmid pXdVexp2 [14] to obtain pXdVexp2-c*ERG10*xd and pXdVexp2-c*POT1*xd, in which each cDNA is located between the ubiquitin promoter and the GPD terminator from *X. dendrorhous*. These plasmids also bear a hygromycin B resistance module for transformant selection.

Wild type strain UCD 67–385 was transformed using linearized plasmids to obtain the transformant strains 385-*ERG10* and 385-*POT1*, in which the gene of interest was inserted by homologous recombination into a non-encoding single copy site named *int* [GenBank: KJ140286] under control of the ubiquitin promoter [14]. A control strain resistant to hygromycin B (385-Vexp2), transformed with the empty plasmid, was also obtained. The correct insertion of each DNA into the target *locus* was confirmed by PCR.

Relative expression for each gene in the overexpression mutants was compared to control strain 385-Vexp2 to determine if indeed there was an increase in transcript levels caused by the additional copy of each cDNA. RT-qPCR was performed on samples taken after 120 h of growth in liquid YM-hygromycin B media to analyze transcript levels of genes *ERG10* and *POT1*. For strain 385-*ERG10,* a 3.2-fold *ERG10* transcript level increment was observed without any significant changes in the levels of *POT1* transcripts. On the other hand, in strain 385-*POT1,* a 4.9-fold increase in *POT1* transcript levels as well as no significant changes in *ERG10* transcripts in relation to the control strain was observed. These results confirm that the integration *locus* is appropriate for inserting additional gene copies for overexpression.

Total carotenoids and sterols were extracted from these strains to determine if gene overexpression had any phenotypic effects, as no differences could be observed to the naked eye. As shown in Table 3, the total amounts of carotenoids and sterols among these strains indeed did not demonstrate any significant differences. Previously, it was reported that *ERG10* (named as *acaT*) overexpression by integration in the rDNA using the *gdh* (glutamate dehydrogenase) promoter led to a slight augmentation in the intracellular astaxanthin content produced by *X. dendrorhous* (approximately 1.3-fold higher than the control strain) [18]. The observed differences in this study may be attributable to several differences in the experimental strategy, including the use of a different integration *locus* [29] and promoter to regulate gene expression [30], gene copy number and the different genetic background of the strain used as the host for gene overexpression.

**Table 3:** Total sterol and carotenoid contents in *X. dendrorhous* thiolase overexpression strains

Strain	UCD 67–385	385-Vexp2	385-*POT1*	385-*ERG10*
Gene overexpression (−fold compared to control)	N/A	1.0 (0.9–1.1)	4.9^*^(3.7–6.4)	3.2^*^(2.2–4.7)
Total sterols (mg/g dry weight)	3.5 ± 0.4	3.8 ± 0.1	3.9 ± 0.2	3.9 ± 0.1
Total carotenoids (μg/g dry weight)	359.0 ± 16.3	401.7 ± 26.8	394.1 ± 15.9	349.7 ± 24.8
% Astaxanthin	75.4 ± 1.2	74.4 ± 0.2	68.7 ± 2.0	73.8 ± 1.4
% Beta-carotene	2.8 ± 0.7	2.6 ± 0.1	4.1 ± 0.9	2.8 ± 0.4
% Phoenicoxanthin	11.6 ± 0.3	10.8 ± 0.7	12.2 ± 0.6	10.9 ± 0.6

Each value corresponds to the average value of three independent samples ± standard deviation. Overexpression data are presented as an average with standard deviation in parentheses. The relative expression of the studied genes in 385-Vexp2 was compared to wild type strain UCD 67–385, and the results indicated no significant differences in the relative expression of these genes; therefore, relative expression for each gene in the overexpression mutants was compared to control strain 385-Vexp2. N/A = Not Analyzed. *p < 0.01, Student’s *T*-test

Our results indicate that the overexpression of either the *ERG10* or *POT1* genes does not lead to significant changes in the amount or composition of carotenoids and sterols in *X. dendrorhous*.

## Conclusions

The *X. dendrorhous ERG10* gene encodes a functional acetyl-CoA C-acetyltransferase (ACAT) that acts in the first step towards mevalonate production, which was supported by a heterologous complementation assay in *S. cerevisiae*. The provided results suggest this gene may be regulated at a transcriptional level through an ergosterol-dependent mechanism. This observation corresponds with previous observations suggesting the possibility of a feedback regulatory mechanism mediated by ergosterol that regulates synthesis of sterols and carotenoids.

A second thiolase-encoding gene (*POT1*) was identified, and its potential involvement in carotenogenesis was tested by constructing *X. dendrorhous* mutants. According to the results, this second gene is not essential for the viability of the yeast and most likely encodes the *X. dendrorhous* 3-ketoacyl-CoA thiolase, which is important for supplying acetyl-CoA for yeast metabolism through β-oxidation of fatty acids. This study provides the first functional evidence that by altering the acetyl-CoA flux in different pathways and cell compartments, the overall synthesis of carotenoids in *X. dendrorhous* is affected.

## Methods

### Strains and culture conditions

All strains and plasmids used in this work are shown in Table 1.

*S. cerevisiae* strains were grown either in YPD media (1% yeast extract, 2% peptone, 2% glucose) or minimal synthetic media SD (0.67% Yeast Nitrogen Base w/o amino acids w/o ammonium sulfate, 0.5% ammonium sulfate, 2% glucose) supplemented with the corresponding amino acids. Geneticin (G418) was used when necessary at a concentration of 50 μg/ml.

*X. dendrorhous* was grown in YM media (0.3% yeast extract, 0.3% malt extract, 0.5% peptone) supplemented with 1% glucose. Hygromycin B at 15 μg/ml or zeocin at 20 μg/ml were supplemented in YM agar (1.5%) plates for transformant selection. All liquid cultures were grown in orbital shakers at 160 rpm at 22 °C for *X. dendrorhous* and at 30 °C for *S. cerevisiae.*

Plasmid propagation was performed in *Escherichia coli* strain DH5α, which was grown in LB media (1% tryptone, 0.5% yeast extract, 0.5% NaCl). For transformant selection, ampicillin was used at a concentration of 100 μg/ml and X-Gal (5-bromo-4-chloro-3-indolyl-β-D-galactopyranoside) at 80 μg/ml.

### Nucleic Acid Extraction

Yeast DNA from *X. dendrorhous* and *S. cerevisiae* was obtained by mechanical cell rupture in 600 μl of TE buffer (25 mM Tris-HCl, 10 mM EDTA, pH 8.0) and 100 μl of 0.5-mm glass beads (BioSpec Products Inc., Bartlesville, OK, USA) using a Mini-beadbeater-16 (BioSpec Products Inc., Bartlesville, OK, USA) for 1 min, followed by centrifugation for 5 min at 4,000 x g to recover the aqueous phase. DNA was extracted by adding 1 volume of a phenol:chloroform:isoamilic alcohol mixture (25: 24: 1, v/v/v), vortexing and centrifuging for 1 min at 20,000 x g. The aqueous phase was recovered and washed with 1 volume of chloroform:isoamilic alcohol (24: 1, v/v) to remove traces of phenol. DNA was precipitated with 1 ml of cold absolute ethanol and incubated at −20 °C for 1 h. Then, it was centrifuged for 10 min at 20,000 x g, and the supernatant was eliminated. The pellet was allowed to dry at 37 °C for 5 min and then suspended in 50 μl of water.

Total RNA was extracted from 2 to 5 ml of *X. dendrorhous* liquid cultures. Cells were harvested by centrifugation, and RNA was obtained using a RiboPure Yeast RNA purification kit (Life Technologies, Carlsbad, CA, USA).

### cDNA synthesis and qPCR

cDNA was synthesized according to the enzyme manufacturer’s protocols in a final volume of 20 μl with 5 μg of total RNA as template using M-MLV reverse transcriptase from Invitrogen (Carlsbad, CA, USA).

Quantitative real-time PCR was performed in an Mx3000P real-time PCR system (Agilent, Santa Clara, CA, USA) using the Sensimix SYBR Green I kit (Bioline, London, UK). Each sample was prepared in triplicate in a final volume of 20 μl using 1 μl of reverse transcription product, 0.25 μM of each primer and 10 μl of kit reaction mixture. To normalize Ct values, the corresponding value for the actin gene of *X. dendrorhous* [GenBank: X89898.1] was employed. In each experiment, the control condition was defined as the untreated culture or the wild type strain. Data were analyzed using the 2^-ΔΔCt^ method, giving an asymmetric distribution of standard deviation values due to the conversion of a exponential process to a linear comparison [31]. Decimal values obtained were converted to negative values according to [31].

### *S. cerevisiae* heterologous complementation assays

All primers used in this work are listed in Additional file 4: Table S1 and were purchased from Integrated DNA Technologies (Coralville, IA, USA). PCR was performed with *Pfu* DNA polymerase (Promega, Madison, WI, USA) using 1 μl of total *X. dendrorhous* RT reaction obtained from the RNA of strain UCD 67–385 to obtain the cDNAs corresponding to each gene. The amplified products were inserted into the plasmid YEp-NP [28]; this plasmid corresponds to a modified YEp-ACT4 plasmid [32] in which a sequence corresponding to 300 bp downstream of the *TDH3* gene of *S. cerevisiae* was inserted into the *Hin*dIII recognition site of the plasmid (Additional file 5: Figure S4, A). Gene orientation in the plasmid was confirmed by colony PCR in a reaction mixture of 25 μl containing 2 U of *Taq* DNA polymerase, *Taq* buffer, 0.2 mM dNTPs, 2 mM MgCl_2_ and 1 μM of each primer.

*S. cerevisiae* was transformed by electroporation. To prepare electrocompetent cells, a 30-ml liquid culture of *S. cerevisiae* that was grown in YPD media overnight at 22 °C was diluted by adding 30 ml of YPD media and incubated for 3 h. Cells at the exponential phase of growth were collected by centrifuging for 5 min at 4,000 x g and washing three times with 40 ml of distilled sterile water. The last wash was performed with 5 ml of 1 M sorbitol. Finally, cells were suspended in 0.2 ml of 1 M sorbitol and divided in 40-μl aliquots, which were stored at 4 °C until use. Before electroporation, 4 μl of plasmid DNA was added to the cells, and the mixture was placed in an electroporation cuvette. The electric pulse conditions were 1.5 kV, 25 μF, and 200 Ω using a GenePulser Xcell ™ (BioRad, Hercules, CA, USA). Cells were suspended in 1 ml of YEP media and incubated at 30 °C for 2 h. Finally, cells were plated on SD plates supplemented with 0.002% uracil and 1% histidine.

For sporulation induction, *S. cerevisiae* transformants were streaked on pre-sporulation agar plates (5% glucose, 0.8% yeast extract, 0.3% peptone, 2% agar) and incubated for 2 days at 30 °C. Cells were transferred to a sporulation agar plate (1% potassium acetate, 2% agar) and incubated at 22 °C until asci were observed by optical microscope (3 to 5 days). To recover the asci from the plate, 1 ml of sterile water was added onto the plate surface, cells were removed using a standard spreader and recovered into an Eppendorf tube. Ascospore isolation was performed using a combined diethyl ether and zymolyase treatment according to [33]. The cell suspension was spread on YEP agar plates supplemented with G418 to confirm the presence of the *S. cerevisiae erg10* ^*−*^ mutant allele. Randomly selected colonies were replica plated on SD agar plates supplemented with uracil and histidine, or with uracil, histidine, lysine and methionine, to sustain all possible auxotrophies in the resulting haploid strains. Then, haploid strains were selected according to their methionine and/or lysine auxotrophy, both of which are heterozygous markers in the parental diploid strain, as auxotrophic strains because these amino acids should be haploid.

### Plasmid construction for *X. dendrorhous* mutation

All plasmids used in this work are presented in Table 1. To eliminate the *ERG10* (Ex: *ERG10A*) and *POT1* (Ex: *ERG10B*) genes from the *X. dendrorhous* genome*,* the plasmids pPHT-ERG10xd and pPHT-POT1xd were constructed (Additional file 5: Figure S4, B). For this, the upstream and downstream regions of each gene of interest were PCR-amplified using genomic DNA from *X. dendrorhous* strain UCD 67–385 as a template. Then, the upstream region was attached to the downstream region using overlap extension PCR [34], introducing a *Sma*I recognition site between them according to the primer design. The fragment was inserted in pBluescript SK at a *Sma*I site to obtain plasmids pBS-PT-ERG10xd and pBS-PT-POT1xd. Each plasmid was digested with *Sma*I to insert either a hygromycin B or zeocin resistance module, which were obtained via digestion from plasmids pMN*-hph* [12] or pIR-zeo [16], respectively. To over-express the *ERG10* and *POT1* genes in *X. dendrorhous*, plasmids pXdVexp2-c*ERG10*xd and pXdVexp2-c*POT1*xd were constructed (Additional file 5: Figure S4, C). The *X. dendrorhous ERG10* and *POT1* cDNAs were amplified from plasmids YEpNPc10xd and YEpNPcPOT1xd, respectively, and were independently inserted at the *Hpa*I site of pXdVexp2 [14].

*X. dendrorhous* electrocompetent cells were prepared from an exponentially growing culture at an O.D._600_ of 2.0 according to [35]. Electroporation was performed using a GenePulser Xcell ™ (BioRad, Hercules, CA, USA), employing a square wave protocol of 5 pulses of 10 ms of 450 V each with 2 ms rest between pulses. For each transformation, 5 μg of linear DNA was used.

### Sterol and carotenoid extraction and RP-HPLC analysis

Sterols and carotenoids were extracted, quantified spectrophotometrically and normalized to the dry weight of the yeast.

Sterols were extracted according to [36]. Briefly, cell pellets were saponified adding 4 g of KOH and 16 ml of 60% (v/v) ethanol/water at 80 °C for 2 h. Then, the mixture was cooled and sterols were extracted using 10 ml of petroleum ether. Total sterols were quantified spectrophometrically at 280 nm using an absorption coefficient of A_1%_ = 11,500. Ether was evaporated and sterols were suspended in 200 μl of methanol and separated by RP-HPLC using a C-18 column and methanol:water (97: 3, v/v) as the mobile phase with a 1 ml/min flux under isocratic conditions. Sterols were visualized with the 280 nm channel, and the elution spectra were recovered using a diode array detector. Standard ergosterol was acquired from Sigma (Saint Louis, MI, USA).

Carotenoids were extracted following [37]. Cell pellets were disrupted using glass beads and acetone for carotenoid extraction. Carotenoids were quantified spectrophotometrically at 465 nm using an absorption coefficient of A_1%_ = 2,100. For carotenoid identification, samples were run in an RP-HPLC using a C-18 column and acetonitrile: methanol:isopropanol (85: 10: 5, v/v/v) as the mobile phase with a 1 ml/min flux under isocratic conditions and compared to standards according to retention time and absorption spectra.

## Additional files

Additional file 1: Figure S1. Protein sequence alignment used for phylogenetic tree construction. The deduced protein sequences for *ERG10A* and *ERG10B* from *X. dendrorhous* were aligned against sequences of diverse thiolases using ClustalW 2.1 and visualized in UGENE. Fully conserved residues are colored in blue. Mitochondrial thiolase/acetyl-CoA C-acetyltransferase: *B. taurus* [NP_001039540.1], *H. sapiens* [BAA01387.1], *C. lupus familiaris* [XP_546539.2], *R. norvegicus* [NP_058771.2], *G. gallus* [NP_001264708.1]. Cytoplasmic thiolase/acetyl-CoA C-acetyltransferase: *S. pombe* [Q9UQW6.1], *B. graminis f. sp. tritici 96224* [EPQ61678.1], *S. cerevisiae* [P41338.3], *N. tabacum* [AAU95618.1], *Z. mays* [NP_001266315.1], *A. thaliana* [Q9FIK7.1]. Mitochondrial thiolase/3-ketoacyl-CoA thiolase: *B. taurus* [NP_001030419.1], *M. musculus* [NP_803421.1], *R. norvegicus* [NP_569117.1]. Peroxisomal thiolase/3-ketoacyl-CoA thiolase: *S. cerevisiae* [CAA37472.1], *Y. lipolytica* [Q05493.1], *G. gallus* [NP_001184217.1], *M. musculus* [NP_570934.1], *E. caballus* [XP_001488609.1], *C. lupus familiaris* [XP_534222.2]. *B. taurus* [NP_001029491.1], *H. sapiens* [NP_001598.1]. *X. dendrorhous* [*ERG10A*]: thiolase encoded by the *ERG10A* gene. *X. dendrorhous* [*ERG10B*]: thiolase encoded by the *ERG10B* gene. (TIFF 8375 kb)
Additional file 2: Figure S2. Pathways involving genes studied in this work. A schematic representation of (A) Sterol and carotenoid synthesis pathways and (B) β-oxidation of fatty acids is shown. The mevalonate pathway is highlighted inside the dashed rectangle. Steps catalyzed by the enzymes encoded by the genes studied in this work (*ERG10* and *POT1*) are highlighted in red. (TIFF 447 kb)
Additional file 3: Figure S3.
*S. cerevisiae* complementation strain growth curves. Strains Sc-*ERG10/erg10* (blue)*,* Sc-ERG10sc (red) and Sc-cERG10xd (green) were grown in YEP media for 48 h with constant agitation at 30 °C. Each point represents the average of three independent cultures. Black bars indicate standard deviation. (TIFF 450 kb)
Additional file 4: Table S1. Primers designed and used for this work. (DOCX 107 kb)
Additional file 5: Figure S4. Scheme of plasmids constructed in this work. Schematic representation of plasmids used for yeast transformation. Selection markers are represented by blue arrows, promoters and terminators, by white arrows; yeast genomic sequences used as a platform for homologous recombination are shown in green and cDNA sequences that correspond to the gene of interest (GOI) are represented by red arrows. Plasmid size in bp is shown considering *POT1* as the GOI. (A) Representation of plasmid YEp-NP, used for heterologous complementation assays, where the cDNA of the gene GOI is represented by a red arrow. (B) Representation of plasmid pPHT-GOI, used to obtain DNA fragments to mutate *X. dendrorhous.* Green arrows represent the positions of the upstream and downstream regions of the GOI. (C) Representation of plasmid pXdVexp2 used for genomic insertion of genes for overexpression in *X. dendrorhous*. Abbreviations; Hygromycin resistance gene (HygR), ampicillin resistance gene (AmpR), *S. cerevisiae LEU2* gene (LEU2). (TIFF 386 kb)

## Abbreviations

ACAA: 3-ketoacyl-CoA thiolase
ACAT: Acetyl-CoA C-acetyltransferase
ATCC: American type culture collection
DRM: Double recombinant method
HMG-CoA: 3-hydroxy-3-methylglutaryl-CoA
HMGR: HMG-CoA reductase
HMGS: HMG-CoA synthase
IPP: Isopentenyl pyrophosphate
MEP: Methyl-D-erythritol-4-phosphate
MVA: Mevalonate
SREBP: Sterol regulatory element binding protein
