# Supplementary figures and images for: Functional characterization of thiolase-encoding genes from Xanthophyllomyces dendrorhous and their effects on carotenoid synthesis
Source: BMC Microbiol. 2016 Nov 21;16:278. doi: 10.1186/s12866-016-0893-2 (PMC5117609; doi:10.1186/s12866-016-0893-2)

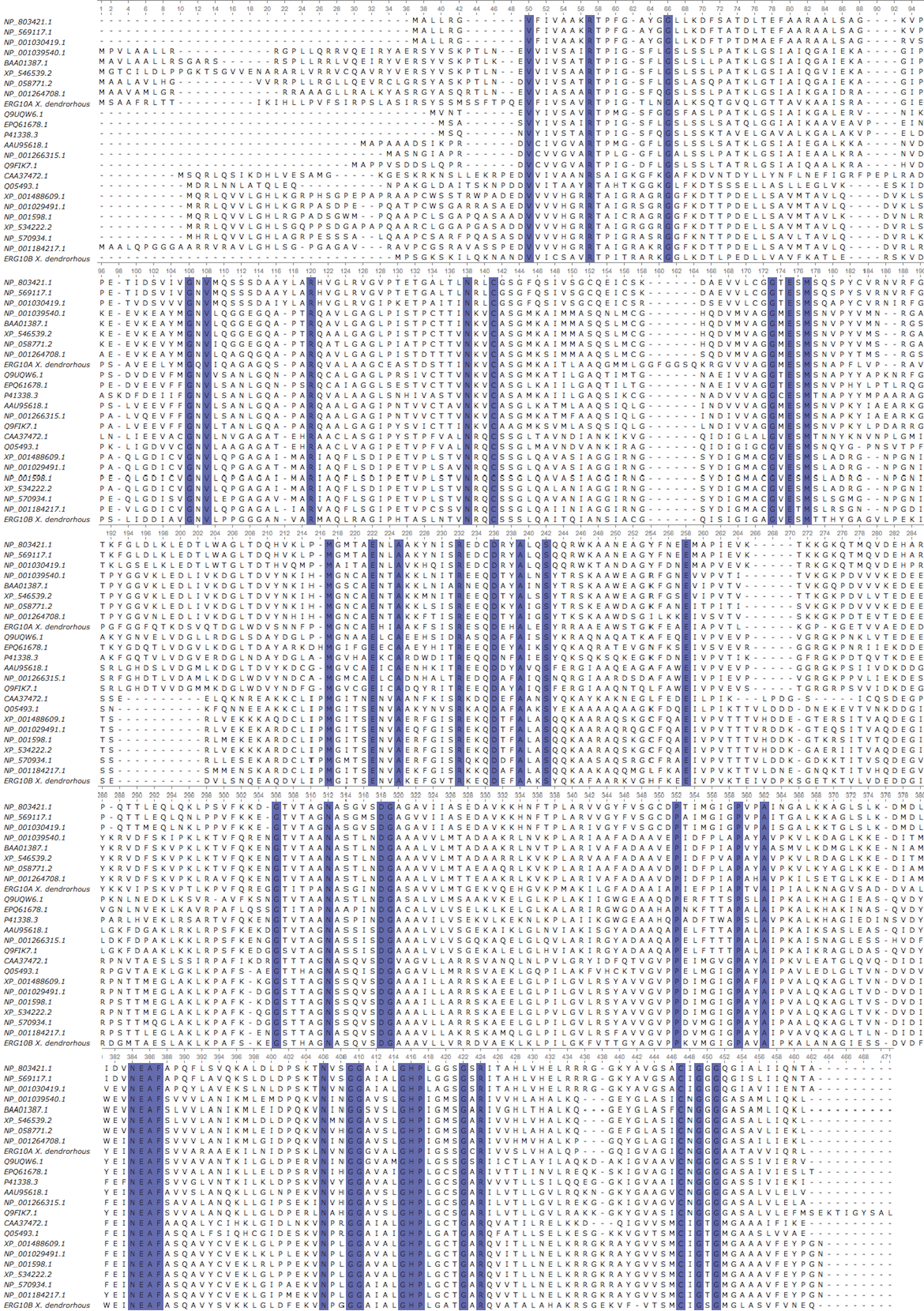

Supplement: Additional file 1: Figure S1. — Protein sequence alignment used for phylogenetic tree construction. The deduced protein sequences for ERG10A and ERG10B from X. dendrorhous were aligned against sequences of diverse thiolases using ClustalW 2.1 and visualized in UGENE. Fully conserved residues are colored in blue. Mitochondrial thiolase/acetyl-CoA C-acetyltransferase: B. taurus [NP_001039540.1], H. sapiens [BAA01387.1], C. lupus familiaris [XP_546539.2], R. norvegicus [NP_058771.2], G. gallus [NP_001264708.1]. Cytoplasmic thiolase/acetyl-CoA C-acetyltransferase: S. pombe [Q9UQW6.1], B. graminis f. sp. tritici 96224 [EPQ61678.1], S. cerevisiae [P41338.3], N. tabacum [AAU95618.1], Z. mays [NP_001266315.1], A. thaliana [Q9FIK7.1]. Mitochondrial thiolase/3-ketoacyl-CoA thiolase: B. taurus [NP_001030419.1], M. musculus [NP_803421.1], R. norvegicus [NP_569117.1]. Peroxisomal thiolase/3-ketoacyl-CoA thiolase: S. cerevisiae [CAA37472.1], Y. lipolytica [Q05493.1], G. gallus [NP_001184217.1], M. musculus [NP_570934.1], E. caballus [XP_001488609.1], C. lupus familiaris [XP_534222.2]. B. taurus [NP_001029491.1], H. sapiens [NP_001598.1]. X. dendrorhous [ERG10A]: thiolase encoded by the ERG10A gene. X. dendrorhous [ERG10B]: thiolase encoded by the ERG10B gene. (TIFF 8375 kb) [file 12866_2016_893_MOESM1_ESM.tiff]

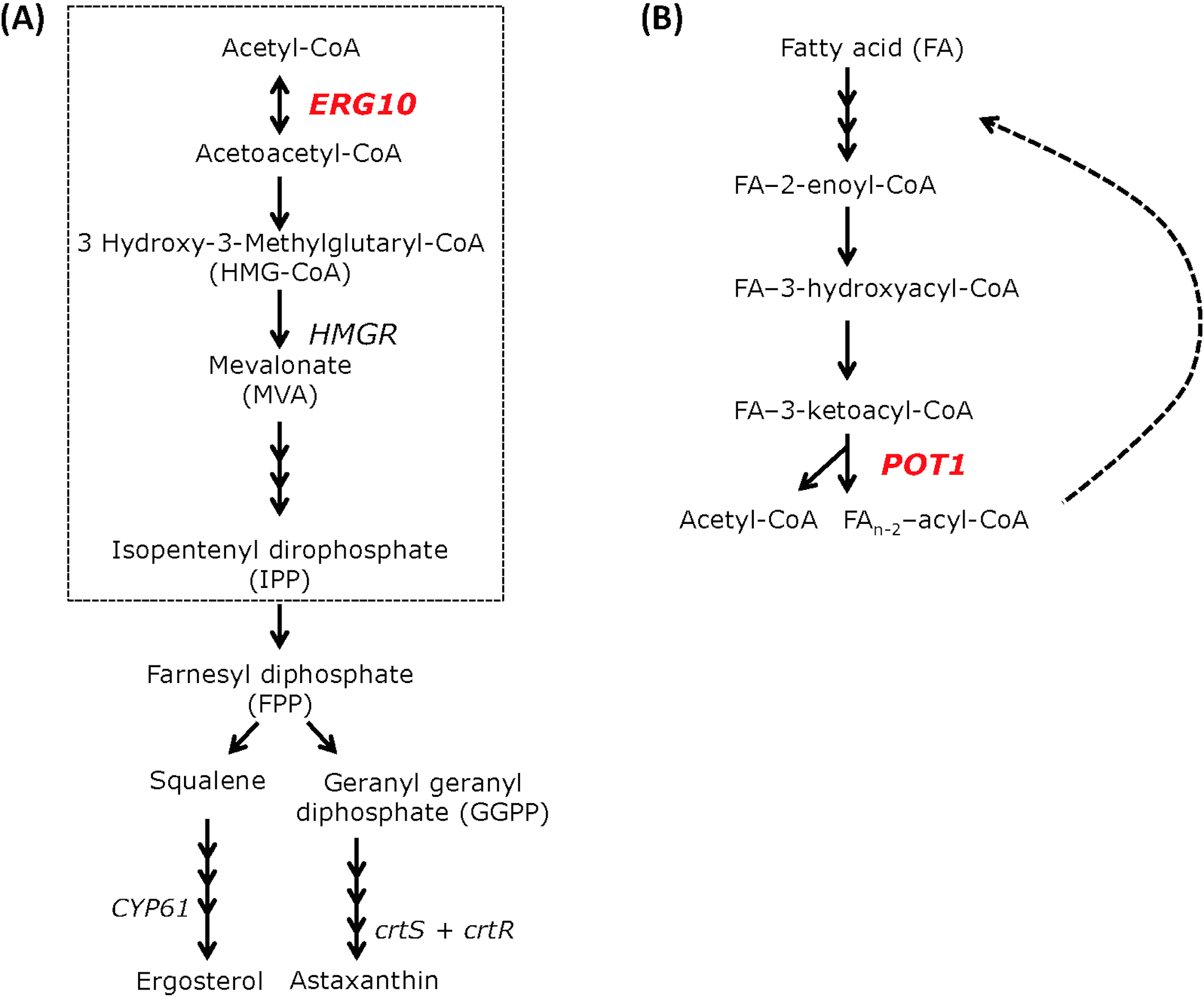

Supplement: Additional file 2: Figure S2. — Pathways involving genes studied in this work. A schematic representation of (A) Sterol and carotenoid synthesis pathways and (B) β-oxidation of fatty acids is shown. The mevalonate pathway is highlighted inside the dashed rectangle. Steps catalyzed by the enzymes encoded by the genes studied in this work (ERG10 and POT1) are highlighted in red. (TIFF 447 kb) [file 12866_2016_893_MOESM2_ESM.tiff]

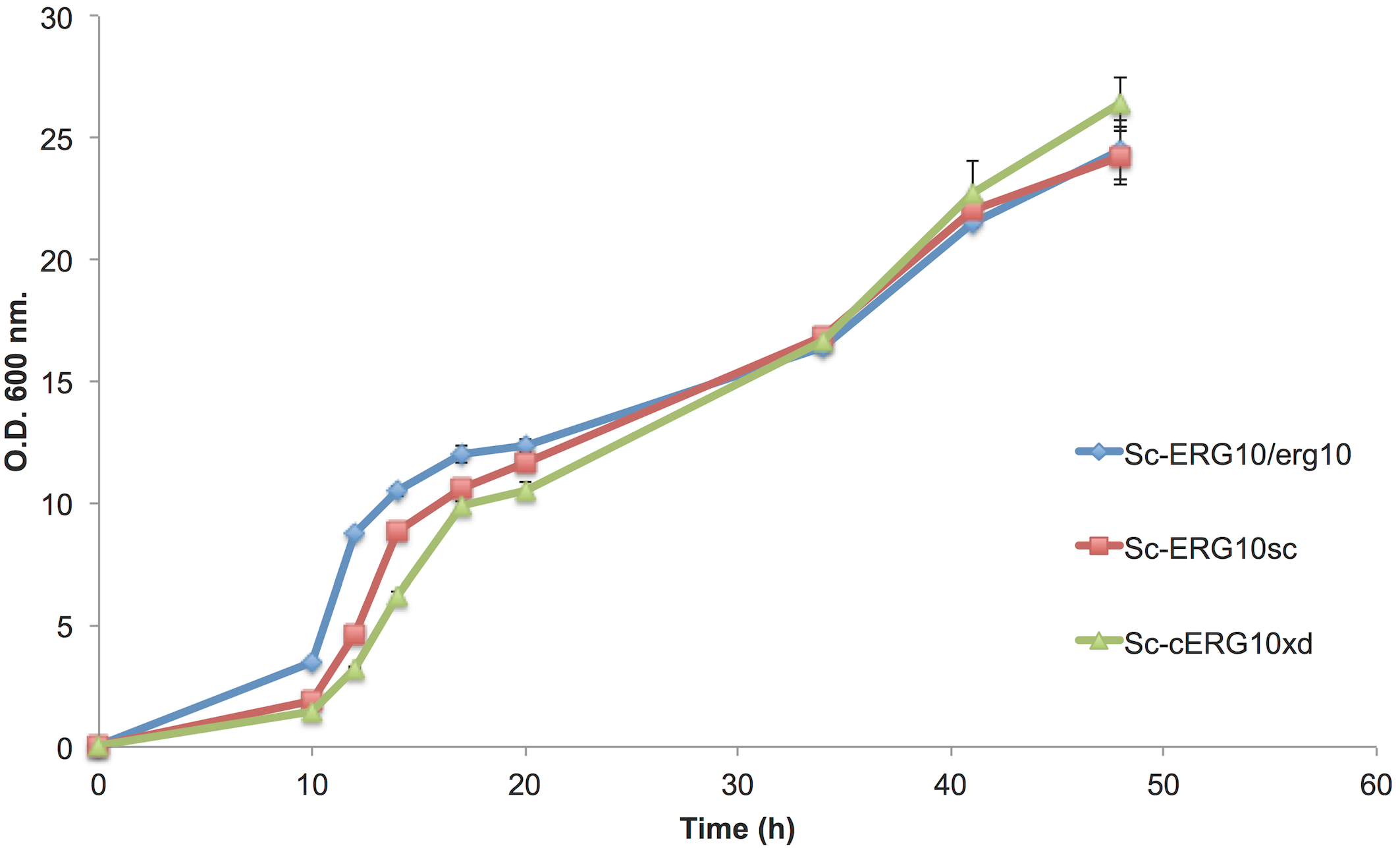

Supplement: Additional file 3: Figure S3. — S. cerevisiae complementation strain growth curves. Strains Sc-ERG10/erg10 (blue), Sc-ERG10sc (red) and Sc-cERG10xd (green) were grown in YEP media for 48 h with constant agitation at 30 °C. Each point represents the average of three independent cultures. Black bars indicate standard deviation. (TIFF 450 kb) [file 12866_2016_893_MOESM3_ESM.tiff]

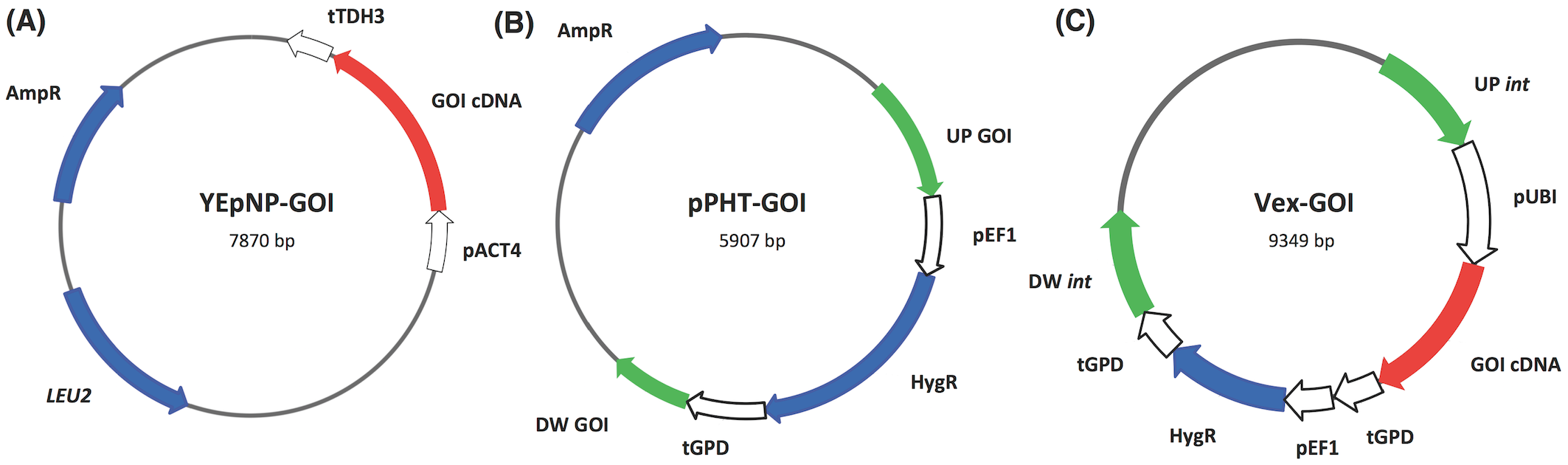

Supplement: Additional file 5: Figure S4. — Scheme of plasmids constructed in this work. Schematic representation of plasmids used for yeast transformation. Selection markers are represented by blue arrows, promoters and terminators, by white arrows; yeast genomic sequences used as a platform for homologous recombination are shown in green and cDNA sequences that correspond to the gene of interest (GOI) are represented by red arrows. Plasmid size in bp is shown considering POT1 as the GOI. (A) Representation of plasmid YEp-NP, used for heterologous complementation assays, where the cDNA of the gene GOI is represented by a red arrow. (B) Representation of plasmid pPHT-GOI, used to obtain DNA fragments to mutate X. dendrorhous. Green arrows represent the positions of the upstream and downstream regions of the GOI. (C) Representation of plasmid pXdVexp2 used for genomic insertion of genes for overexpression in X. dendrorhous. Abbreviations; Hygromycin resistance gene (HygR), ampicillin resistance gene (AmpR), S. cerevisiae LEU2 gene (LEU2). (TIFF 386 kb) [file 12866_2016_893_MOESM5_ESM.tiff]
