# Supplementary material for: Functional characterization of thiolase-encoding genes from Xanthophyllomyces dendrorhous and their effects on carotenoid synthesis
Source: BMC Microbiol. 2016 Nov 21;16:278. doi: 10.1186/s12866-016-0893-2 (PMC5117609; doi:10.1186/s12866-016-0893-2)
Supplement: Additional file 4: Table S1. — Primers designed and used for this work. (DOCX 107 kb) [file 12866_2016_893_MOESM4_ESM.docx]

**Table S1.-** Primers designed and used in this work.

| **Primer** | **Sequence 5'-3'** | **Target** |
| --- | --- | --- |
| pot1_genFW | atgccatctggaaagtccaag | *X. dendrorhous POT1* gene. |
| pot1_genRV | tcactgctcattcacaaacacc | *X. dendrorhous POT1* gene. |
| Thio2_fw | atgtctgccgccttccgtc | *X. dendrorhous ERG10* gene. |
| Thio2_rv | ttagagcctctggatgacgac | *X. dendrorhous ERG10* gene. |
| erg10sc_F | tctagatgtcacagaacgtttatattg | *S. cerevisiae ERG10* gene |
| erg10sc_R | aagcttttaaggcagttgatgtttgct | *S. cerevisiae ERG10* gene |
| M13.Fw.FP | tgtaaaacgacggccagt | M13 Region from vector YEpNP. |
| M13.Rv.PUC-40 | ggaaacagctatgaccatg | M13 Region from vector YEpNP. |
| KanMX4.F2 | ttgtatgggaagcccgatg | *kanMX* gene (G-418 resistance cassette). |
| KanMX4.R2 | gatcctggtatcggtctgc | *kanMX* gene (G-418 resistance cassette). |
| H.F | atgaaaaagcctgaactcacc | *hph* gene (hygromycin B resistance cassette)*.* |
| H.R | ctattcctttgccctcggac | *hph* gene (hygromycin B resistance cassette)*.* |
| pot1_PR_Fw | gtggaaaggaaaggacgaacg | *X. dendrorhous POT1* upstream region |
| pot1_PR_Rv | tgaccacatcgttcgcgttct | *X. dendrorhous POT1* upstream region |
| pot1_T_Fw | ctgaagcttccgatcttgggc | *X. dendrorhous POT1* downstream region |
| pot1_T_Rv | caaagaagcccttcgtcccac | *X. dendrorhous POT1* downstream region |
| erg10_PR_Fw | gctcacttgaccaagcggc | *X. dendrorhous ERG10* upstream region |
| erg10_PR_Rv | ttattaggccagcaggcggc | *X. dendrorhous ERG10* upstream region |
| erg10_T_Fw | ctactgctgtcgtcatccag | *X. dendrorhous ERG10* downstream region |
| erg10_T_Rv | ggtgaggagatagttactgac | *X. dendrorhous ERG10* downstream region |
| pot1_Rv_P_Sma_T | ggaagcttcag*cccggg*tgaccacatcgttcgcg | To construct pBS-PT-*POT1xd*: 11 bases of the *X. dendrorhous POT1* gene downstream region (underlined), *Sma*I restriction site (italics) and 17 bases of the *X. dendrorhous POT1* gene upstream region*.* |
| pot1_Fw_T_Sma_P | gaacgatgtggtca*cccggg*ctgaagcttccgatcttgg | To construct pBS-PT-*POTxd*: 14 bases of the *X. dendrorhous POT1* gene upstream region (underlined), *Sma*I restriction site (italics) and 19 bases of the *X. dendrorhous POT1* gene downstream region*.* |
| erg10_Rv_P_Sma_T | cgacagcagtag*cccggg*ttattaggccagc | To construct pBS-PT-*ERG10xd*: 12 bases of the *X. dendrorhous ERG10* gene downstream region (underlined), *Sma*I restriction site (italics) and 13 bases of the *X. dendrorhous ERG10* gene upstream region*.* |
| erg10_Fw_T_Sma_P | ctggcctaataa*cccggg*ctactgctgtcgtcatc | To construct pBS-*ERG10xd*: 12 bases of the *X. dendrorhous ERG10* gene upstream region (underlined), *Sma*I restriction site (italics) and 17 bases of the *X. dendrorhous ERG10* gene downstream region*.* |
| pEFrev0 | tttgaagctgttcgagatag | *X. dendrorhous* *TEF* promoter (hygromycin B resistance cassette) |
| gpdTF | acggttctctccaaaccctc | *X. dendrorhous* *GPD* terminator (hygromycin B resistance cassette) |
| GPDHtRev | atcatgagagatgacggag | *X. dendrorhous* *GPD* terminator (hygromycin B resistance cassette) |
| PEFForEV | gatatcggctcatcagccgacagtt | *X. dendrorhous* *TEF* promoter (hygromycin B resistance cassette) |
| Erg10upF | gcactgctaagcctactcg | 79 bp upstream of *S. cerevisiae ERG10* gene |
| Erg10dwR | ccg aat gtt gga aca ggt gct | 76 bp downstream from *S. cerevisiae ERG10* gene |
| UP_erg10_Xd | ccatagcaaccgtcatctc | Upstream *X. dendrorhous ERG10* gene |
| DW_erg10_Xd | ctcttggatgttacgaaagg | Downstream *X. dendrorhous ERG10* gene |
| UP_pot1_Xd | agcagtgttcctcaccttccg | Upstream *X. dendrorhous POT1* gene |
| DW_pot1_Xd | ccttccaaagctcatccaaag | Downstream *X. dendrorhous POT1* gene |
| Ext-pre-no-cod-F | gttctttgcgtcccccaaac | Upstream *X. dendrorhous locus int* [Genbank: KJ140286] |
| Ext-post-no-cod-R | ctggccctcctgtggtctgtc | Downstream *X. dendrorhous locus int* [Genbank: KJ140286] |
| nocod_fw_pre | ccaaaatccagacgcaccctc | Non coding sequence of *X. dendrorhous locus int* [Genbank: KJ140286] |
| nocod_rv_post | ggtctgggggtgatagtagatg | Non coding sequence of *X. dendrorhous locus int* [Genbank: KJ140286] |
| P.ubi.fw | tctatggactaagtgtccgacc | *X. dendrorhous* *UBI* promoter (overexpression cassette) |
| T.gpd.R | atgagagatgacggagatgat | *X. dendrorhous* *gpd* terminator (overexpression cassette) |
| **RT-qPCR** |  |  |
| mcrtRR-RT-F | ctgggaaacaagacctacga | *X. dendrorhous crtR* gene cDNA |
| mcrtRR-RT-R | ggaacctcggttacgacaaa | *X. dendrorhous crtR* gene cDNA |
| mActF-RT | ccgccctcgtgattgataac | *X. dendrorhous ACT* gene cDNA |
| mActR-RT | tgaccaaggtaggagtcctt | *X. dendrorhous ACT* gene cDNA |
| RT_pot1_fw | tccctcatactgcgtctttg | *X. dendrorhous POT1* gene cDNA |
| RT_pot1_rv | tggttggagagcacatcttc | *X. dendrorhous POT1* gene cDNA |
| RT_erg10_fw | aggcttcgaccgattctact | *X. dendrorhous ERG10* gene cDNA |
| RT_erg10_rv | aggaaaggagcgttggacat | *X. dendrorhous ERG10* gene cDNA |
| ADH_RT Fw A | tgacccagtcggaatcaagt | *X. dendrorhous ADH1* gene cDNA |
| ADH_RT Rv A | taacgtgggcagcgtaaga | *X. dendrorhous ADH* gene cDNA |
| mcrtSF-RT | atggctcttgcagggtttga | *X. dendrorhous crtS* gene cDNA |
| mcrtSR-RT | tgctccataagctcgatcccaa | *X. dendrorhous crtS* gene cDNA |
| grg2real FW1 | catcaagacctctgtcaccaac | *X. dendrorhous GRG2* gene cDNA |
| grg2real RV1 | ttggcgtcagacgaggact | *X. dendrorhous GRG2* gene cDNA |
